# Healthcare Providers’ Experiences Accessing Real-Time Virtual Support: Informing More Equitable and Inclusive Healthcare Access in British Columbia’s Rural, Remote, First Nations, and Other Indigenous Peoples and Communities

**DOI:** 10.1177/08404704251405215

**Published:** 2026-02-06

**Authors:** Hollis Owens, Nazia Nadir Shah, Michelle Lin, Rochelle Chauhan, Joan Assali, Amrit Bhullar, Kurtis Stewart, Kendall Ho, Anne Lesack, Erika Pritchard, Helen Novak Lauscher

**Affiliations:** 1 8166 Department of Emergency Medicine, The University of British Columbia, Vancouver, British Columbia, Canada; 2 590705 Rural Coordination Centre of British Columbia, Vancouver, British Columbia, Canada

## Abstract

Healthcare Providers (HCPs) serving Rural, Remote, First Nations, and other Indigenous (RRFNI) communities face unique challenges in delivering longitudinal care due to geographic isolation. The Real-Time Virtual Support Services (RTVS) network aims to improve equitable access to healthcare and provide collegial support for HCPs in RRFNI communities across British Columbia. The objective of this study was to understand HCPs’ experiences with RTVS and identify improvement areas. Data were collected through semi-structured interviews with HCPs that were recorded, transcribed, and openly coded. Twenty HCPs using RTVS were interviewed during 2022-2023. The constant comparative method was used to develop themes. Themes focused on RTVS’s benefits and outcomes including increased clinical confidence, reduced provider anxiety, respectful and collegial support, reduced administrative burden, and recruitment and retention support. Challenges included occasional service disruptions and limited Wi-Fi availability. These findings provide in-depth and contextualized feedback informing the development of RTVS.

## Introduction

### Background and Context

Rural, Remote, First Nations, and other Indigenous (RRFNI) communities across Canada experience significantly worse health outcomes with higher rates of preventable and treatable mortality than their non-rural counterparts.^1^ With only 8% of physicians practicing in RRFNI communities across Canada despite 18% of the total Canadian population living in these areas,^2^ access to healthcare in RRFNI communities is lacking, posing a challenge for individuals living in these communities.^3^

General Practitioners (GPs) working in RRFNI communities noted feelings of exhaustion, highlighting that working in remote conditions required excessive demands, leaving little time for their personal lives.^4,5^ This is consistent with GPs experiencing high levels of burnout globally.^6^ The effects of burnout go beyond Healthcare Providers (HCPs) satisfaction; a recent meta-analysis showed that burnout in nurses was associated with lower quality of healthcare provided and worse outcomes for patient safety.^7^ Burnout is also a factor to consider when addressing the issue of provider retention in RRFNI communities, as it further increases the risk of attrition of HCPs. Outside of professional burnout, HCPs located in RRFNI communities experience other challenges affecting retention. HCPs reported a lack of resources, an insufficient number of physicians leading to increased service demands, fewer opportunities for professional support, and a heightened sense of responsibility.^4,5^ Workforce support and physician recruitment and retention are some of the challenges that have persisted in BC since the 2000s. The 2001–2002 annual Ministry of Health (MoH) report documented significant difficulties in maintaining adequate physician coverage in rural/remote areas in British Columbia (BC), necessitating the creation of targeted support programs.^8^ Mohr et al. (2006) evaluated one such early-generation telehealth program that was implemented in northern and remote communities of BC as a means to improve access and continuity of care.^9^ Their findings underscored enduring systemic issues, including geographic and access barriers, healthcare workforce shortages, and infrastructure inequities. Fast forward 14 years, and Real-Time Virtual Support (RTVS) was implemented as a provincial network of virtual services in 2020 aimed at improving equity of access, particularly for rural and remote populations.

### Real-Time Virtual Support (RTVS) Network

To address the HCP shortage in RRFNI communities, hybrid care (combining virtual and in-person services that are fully integrated with a patient’s in-person ecosystems of team-based care) has been identified as a solution for some of the challenges that HCPs practicing in these communities face. A 2020 scoping review conducted by Leblanc et al. found an overwhelmingly positive response to telehealth interventions, with benefits including a decrease in travel and wait times for patients, a reduction in cost, and increased access to health services. The RTVS network in BC, a collaboration between BC Ministry of Health, Rural Coordination Centre of BC (RCCbc), the First Nations Health Authority (FNHA), and UBC Digital Emergency Medicine (DigEM), was established in 2020 to improve health equity in RRFNI communities.^10,11^

RTVS uses a Learning Health Systems (LHS) approach towards quality improvement.^12^ A LHS operates through a continuous loop that begins with forming a multistakeholder learning community, followed by collecting system performance data, analyzing it to identify improvement opportunities, and implementing interventions based on that analysis.^13^ The RTVS LHS is an iterative growth process for health improvement that supports RTVS’s aims to improve healthcare access in RRFNI communities. RTVS’s LHS emphasises engaging stakeholders and collaborative ecosystems that brings together HCPs, community members, and researchers through partnerships with RCCbc, MoH, and FNHA.

**Figure 1.**
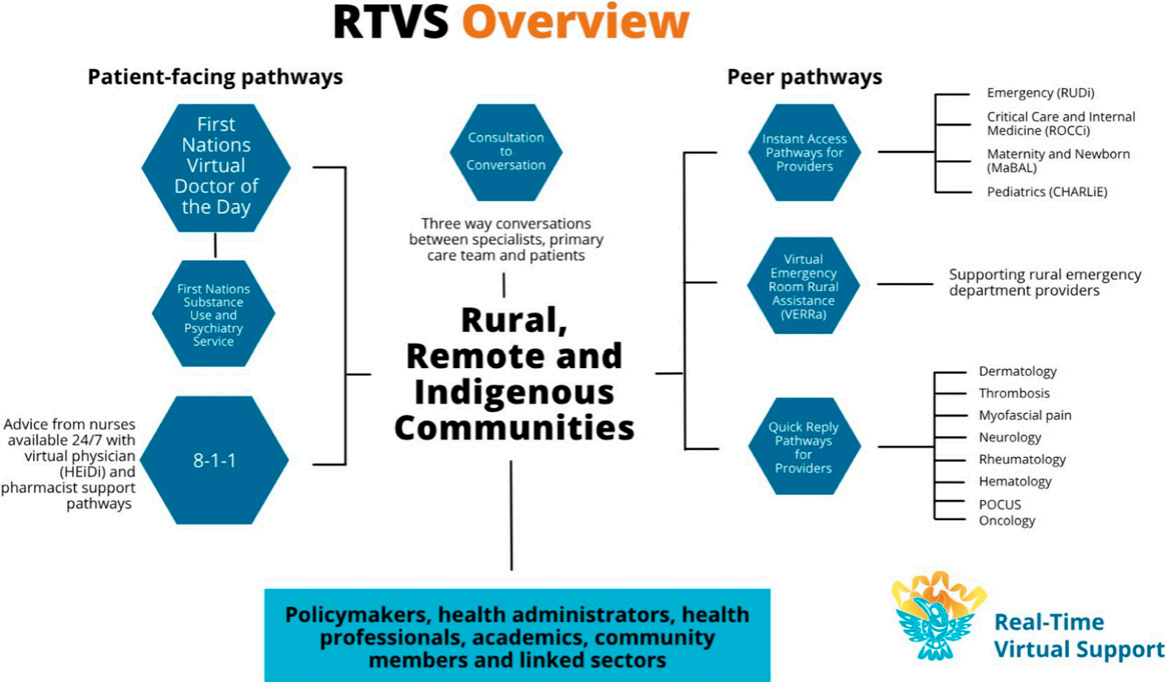
Overview of RTVS Pathways

The RTVS initiative offers two main types of virtual healthcare pathways: provider-to-patient-facing pathways and peer-to-peer pathways. The provider-to-patient-facing pathways are direct care services from a virtual provider to a patient, ensuring rural and remote patients can access timely, culturally safe, and appropriate care. The peer pathways provide real-time advice, guidance, and mentorship from specialists or experienced peers to rural, remote, and Indigenous frontline HCPs. Rural providers in RRFNI communities can access 24/7 clinical support through RTVS Instant Access peer pathways via Zoom or telephone, or specialized support Monday to Friday through Quick Reply peer pathways via Zoom or telephone. See Figure 1 for an overview of all RTVS pathways.^14^

Among the instant access peer pathways are the Maternity and Babies Advice Line (MaBAL), Child Health Advice in Real-Time Electronically (CHARLiE), and Rural Urgent Doctor in-aid (RUDi), which were the focus of this study.

MaBAL calls are answered by full-service family physicians with expertise in maternal and newborn care who offer immediate clinical support and consultation during antepartum, labour, delivery, and post-partum periods of pregnancy. MaBAL also provides coaching sessions for nurses to enhance their confidence and competence in supporting maternity cases in rural settings.^15^ CHARLiE provides paediatric specialist advice, comprehensive consultation, support for neonatal and paediatric resuscitation and stabilization via pediatricians, paediatric emergency physicians, and paediatric intensivists.^16^ RUDi is staffed by emergency physicians and full-service physicians with experience in emergency medicine training. RUDi ensures that healthcare teams in RRFNI communities have access to timely and expert emergency support, real-time consultation, and support with health system navigation.^17^

### Study Objectives

A previous quality improvement study^18^ explored the perspectives of Virtual Providers (VPs) staffing the RTVS pathways, highlighting the importance of RTVS pathways as well as the long-term sustainability of the program. This study builds on VPs’ understanding of the RTVS program’s impact on health delivery in RRFNI communities and helps understand rural and remote HCPs’ experiences with RTVS virtual peer pathways for improving RRFNI’s access to care.

## Methods

This study describes the qualitative methods used to evaluate the RTVS peer pathway services through semi-structured interviews from the larger mixed methods evaluation of RTVS in 2022-23.^19^

### Participant Recruitment

Participants were recruited through RTVS contact lists, partner referrals, and snowball sampling. In total, 36 rural/remote HCPs were contacted to participate in the interviews, of whom 20 were interviewed. Inclusion criteria included participants having used either MaBAL, CHARLiE, and/or RUDi RTVS peer pathways (see Figure 1). Ethics was not required after consulting The University of British Columbia (UBC) behavioural research ethics board which provided an ethics waiver. The UBC screening checklist identified the study as a quality improvement activity not subject to institutional ethical review as defined under Article 2.5 of the Tri-Council Policy Statement.^20^

### Data Collection

The semi-structured interviews were 30 to 60 minutes in length and held over Zoom. With the participants’ consent, interviews were recorded and professionally transcribed. The interview guide was co-developed with FNHA, MoH, RCCbc, and UBC DigEM and asked providers about their experience with the RTVS service and areas for improvement.

### Analysis

Following transcription of the interviews, we used NVivo 12 to analyze and code the 20 interview transcripts that totalled 136 pages of single-spaced data. Two evaluation team members coded two interview transcripts separately with an open coding method to describe unique units of data in the transcripts. Once coded, the codebook was developed in working group meetings by reviewing and coming to a consensus on the codes used in these two transcripts. The developed codebook was used to code the remaining transcripts by five team members and was regularly updated to accommodate additional codes in the transcripts. Following the completion of coding, themes were iteratively developed during weekly working group meetings using the constant comparative analysis method.^21^ Codes were iteratively reviewed and discussed by coders and working group team members to understand the relationships across the codes to develop the overarching themes Trustworthiness strategies used included reflexivity through a researcher’s journal and regular working group meeting discussions, audit trails and documentation of the evaluation process, and engaging with team members and partners to review findings.^22^

## Findings

### Participants

The 20 participants included 12 physicians, 7 nurses, and 1 midwife. The nurses interviewed were remote nursing certified, practice consultants, and a senior clinical nurse. Overall, rural and remote HCPs’ practice locations included First Nation communities, nursing stations, family practices, and hospitals. The length of practice for the interviewed nurses and midwife ranged from under 1 year to over 8 years of practice. Three of the nurses worked in communities with Rural Subsidiary Agreement Designation A (RSA-A) as defined by the MoH as the most isolated communities in BC.^23^ The remaining nurse participants and midwife did not specify their RRFNI community with one of these nurses describing her community as very remote and only accessible by plane or boat. The length of practice for the physician participants ranged from under 1 year to over 20 years. Eight of the physician participants worked in communities with RSA designation A; 1 worked in a community with RSA designation B; and the remaining RRFNI communities were not specified. Participants’ reasons for calling RTVS included: clinical decision support, reassurance, case outside their practice scope, assistance with transport, emergency care, and prescription extension. Modalities for accessing RTVS included phone, video (iPad), and chat.

### Themes

**Figure 2.**
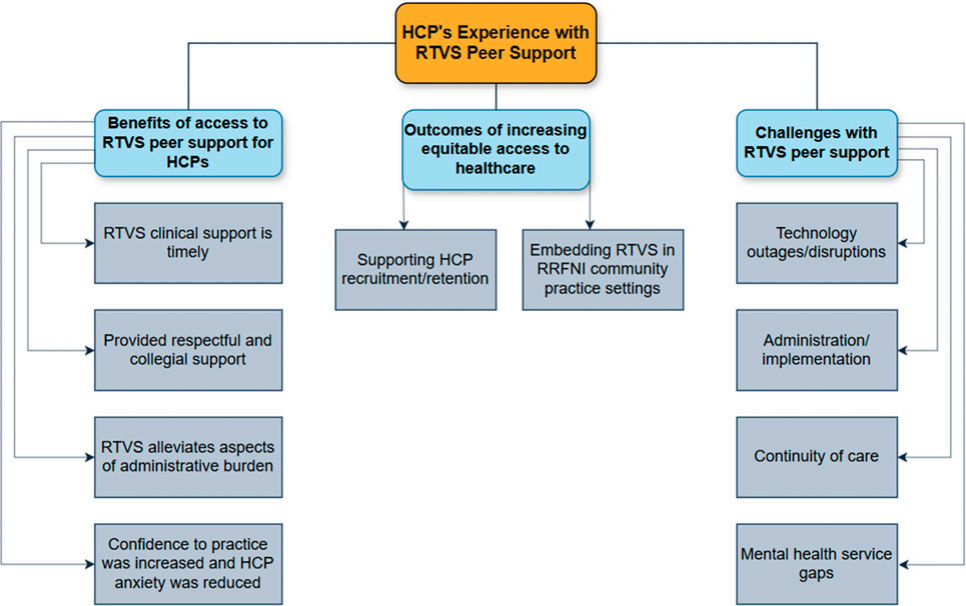
Overview of rural/remote HCP themes describing their experience with RTVS peer support

HCPs’ experience accessing and using RTVS peer support in their clinical practice is summarized into the following categories (see Figure 2): (1) Benefits of accessing RTVS peer support, (2) Outcomes of increasing equitable access to healthcare in RRFNI communities, and (3) Challenges with RTVS peer support. The subsequent sections will elaborate on the themes identified within these three categories.

#### Benefits of Access to RTVS Peer Support for HCPs

##### Theme 1

###### RTVS Clinical Support is Timely

The timeliness of accessing RTVS physician support was discussed frequently by rural/remote HCPs as an advantage in contrast to using traditional methods, such as calling a busy Emergency Department in a large or urban centre and facing potential judgement from an urban peer who may not understand their rural/remote clinical context. One registered nurse and site coordinator explained that having access to RTVS’s timely support also improved quality of care because the on-call RTVS providers have dedicated time to provide clinical support, as one nurse explained:

[W]hat used to happen is we would have to call...a very busy Emergency Department.... get put in the cue, we might have to wait an hour, two hours, three hours, depending on the acuity of the situation. When they did end up calling you back...they did not have a lot of time for you. It’s all understandable. This is just the services that they have... now feel like I have the time to consult for a lot of things that I would never have called for before. So, it’s not just the timing issue, there’s also a quality of care that has significantly improved. (Participant #17)

Findings suggest that RTVS’s dedicated peer support uniquely improves wait times for receiving clinical support in RRFNI communities.

##### Theme 2: RTVS Provided Respectful and Collegial Support Through “How Can I Help?”, Improving Upon the Traditional “Why Did You Call?”

Most HCPs discussed that a main strength of RTVS was the respectful and collegial support provided by VPs, including their characteristics as excellent communicators through a coaching approach and knowledge of the RRFNI practice setting and resources available. Nursing rural/remote HCPs frequently noted that VPs were also respectful and knowledgeable of the remote nursing scope of work. One community health nurse stated that VPs’ understanding of rural/remote and Indigenous communities as well as their respect for the population where the HCPs are working ultimately leads to the provision of culturally safe care for patients:

The doctors [VPs] have a good understanding of the communities that we're working for and it feels like they have a respect for the population, and so, a lot of the more local doctors, there will be a lot of racism that I feel when I'm trying to treat patients, and the RUDi doctors have all been like fairly non-judgmental, and very open to exploring alternative treatments and things like that that maybe fit better with a lifestyle plan and I really appreciate that. (Participant #1)

HCP end-users of RTVS consistently described their interactions with VPs as culturally safe for their context and patients.

In addition, VPs’ approach to coaching was also noted by some rural/remote HCPs as a strength of RTVS, as stated by one nurse:

I think communication is really good with the RTVS physicians [VPs] too, like if they don’t understand something they’re more than willing to ask can you elaborate on that or if my assessment is lacking in an area they just ask if I can perform some of these assessments and with it being virtual that’s definitely a strength so they’re able to watch and coach me as I perform these tests like, so that I get the most accurate result possible so that’s a very helpful feature. (Participant #16)

The respectful and collegial support provided by RTVS VPs was described by some participants as ultimately increasing accessibility to care as explained by a physician:

...having the opportunity to go chat with...a friendly colleague and somebody who has the experience and knowledge who has been there...to review a case with you and reassure you...it really builds your confidence, helps you navigate the system and ultimately provides the best possible care for the patients who are in remote areas and it can save them a trip, which in our case would be a 2-hour drive on an icy road to [community name]. (Participant #19)

Findings suggest that RTVS is staffed by VPs who are trained in and practice cultural safety, are excellent communicators, and are friendly, which supports patient access to care in their home community.

##### Theme 3: RTVS Alleviates Aspects of Administrative Burden around Documentation, Follow-Up, and the Patient Transfer Network (PTN)

A substantial benefit of RTVS drawn from the data was its ability to support rural/remote HCPs when transport was needed, including coordinating with the PTN or avoiding transport when not needed. Most providers expressed that RTVS coordinating with PTN helps improve patients’ care by giving the HCP more time to provide hands on care for their patient, as this rural family physician stated:

One of the things that I find really helpful from RTVS in my experience is the kind of co-management piece where sometimes if we need to call PTN or arrange a transfer for a patient, they can actually make those calls for us or help us with those calls while we’re dealing with the actively sick patient and it keeps us hands-on the patient more and improves care that way. (Participant #18)

In addition to RTVS coordinating with PTN, a few HCPs interviewed also described that receiving a faxed summary from the VP was beneficial for documentation especially when there is a resuscitation or code for a patient as one rural physician stated:

I really like the documentation aspect...after having a call with someone [RTVS VP] so in the case of the code it was great cause it was like having someone who was documenting for us on the other side virtually and then...the physician sent over a report shortly after within like a day. (Participant #9)

Findings suggest that RTVS is essential to support rural/remote HCPs with PTN coordination and provide documentation to enhance patient care in their community.

##### Theme 4: Confidence to Practice Was Increased, and HCP Anxiety Was Reduced Through RTVS Peer Support

All HCPs interviewed also described that having the instant support of an RTVS VP helped them not to feel alone in their practice, resulting in feelings of increased confidence to practice in rural and remote settings. For example, one physician explained:

Having a service like this [RTVS]...that more experienced colleague or specialist in your back pocket...really increases your confidence to practice in those [remote] settings...even if you know the other physicians in town are not available which could definitely happen, I still don’t feel like I’m alone because I have that service...it just makes me a lot more confident. (Participant #5)

Most HCPs interviewed felt that having access to RTVS helped to reduce their anxiety levels associated with practicing in a rural/remote community. A rural/remote registered nurse and site coordinator said that having support from RTVS in their practice reduced their anxiety levels, affecting their whole life and practice:

To have somebody that can back you up in an instant is just like our anxiety levels generally have gone down. So, it’s really affected not just the way we practice but it’s affected our whole life, really, yeah, pretty major thing. (Participant #17)

Findings suggest that the impact of feeling more confident in clinical practice through feeling supported by RTVS translated to increased provider satisfaction and ultimately improved patient and family satisfaction for some participants as discussed by one rural physician:

Feeling better supported, feeling more confident in my diagnosis...translates to better satisfaction for me as a provider...when I am more satisfied and more confident...that can translate to how the patients or the families feel. They feel better cared for knowing that there isn’t a whole lot of uncertainty and that we’re moving forward in a positive way. (Participant #6)

Accessing RTVS peer support to speak with an experienced colleague helped HCPs feel more confident to practice and reduced isolation and anxiety, ultimately improving provider and patient satisfaction.

#### Outcome of Increasing Equitable Access to Healthcare

##### Theme 1: Supporting HCP Recruitment/Retention in RRFNI BC Communities

A core finding shared in most interviews was the ability of RTVS to provide access to high-quality care for patients living in RRFNI communities in BC, as shared by most participants through increasing recruitment and retention of providers, decreasing clinical isolation and anxiety, and encouraging learners to work in a rural community, as explained by one family physician:

They [RTVS]...have filled a gap… they’ve increased accessibility to higher level of care knowledge, lowered the barrier and by doing that they contribute to the retention and recruitment to rural areas... So having RTVS I think allows people to take away a little bit of that anxiety and might allow people to be more willing to come out. (Participant #15)

Nurses also frequently shared that while working in remote nursing stations having RTVS while on call as the only provider in the community for an emergency case, such as a snowmobile accident or heart attack, was particularly beneficial to relieve their anxiety and support their retention in that community.

Several HCPs shared that receiving clinical support from RTVS increases recruitment of HCPs by improving the HCPs’ sense of safety to practice in a rural community and the resources available, as illustrated by an HCP:

I feel like if I didn’t have CHARLiE available I would really consider whether I want to practice in a rural community or not because I would probably feel unsafe in some situations or like I don’t have the resources I need and that’s not safe for me professionally or for my patients. (Participant #3)

This supports the finding that access to RTVS fills a gap that exists for rural/remote HCPs to receive clinical support that helps them feel more confident and safer to practice in an RRFNI community enhancing recruitment and retention.

##### Theme 2: Embedding RTVS in RRFNI Community Practice Settings

Most nursing participants also described that RTVS has become embedded in their practice as participant 14, who is a rural nurse, stated, “I don’t think I could go back to practicing without having you guys.” RTVS has also become the standard of practice in some RRFNI communities, with some patients asking if the HCP is going to consult RTVS for their appointment. Another rural nurse stated:

...people notice in the community, we talk about it [RTVS], it’s sort of becoming the standard now, right, like “well aren’t you gonna call that doctor on the screen?” I’m like “well we don’t need to this time - I can handle this.” (Participant #17)

This also speaks to the importance of RTVS being integrated into a hybrid care mode in a community where virtual care is able to augment and support the in-person HCP working in that community.

#### Challenges With RTVS Support

##### Theme 1: Technology Outages/Disruptions

Despite the benefits that end-user HCPs experienced with RTVS, a few participants also described the challenges they encountered with RTVS peer support. Challenges experienced with technology/connectivity were related to the availability of high-speed Internet in some remote communities, causing disruptions during RTVS video calls or with the use of telehealth carts, as stated by a physician:

The only pitfall or negative thing has been the technology, part of it that’s been at times challenging...especially when we’re running a code and it’s a bit spotty and it’s hard to like hear what we’re telling them or vice versa in those moments is where the Zoom call just drops...that can be frustrating but I think that just applies to any technology in general. (Participant #9)

A remote certified nurse explained her experience with technology disruptions as “...in the [more remote] nursing stations the internet is so poor that unfortunately the Telehealth Carts don’t work…” (Participant #13). However, despite these challenges, these participants expressed the value of RTVS and would access support via phone to help overcome Internet connectivity.

##### Theme 2: Administration/Implementation

Challenges related to the administration and implementation of RTVS included longer wait times and the pathway not answering as described by two participants. While this happened infrequently, it could be very challenging as a physician discussed:

...When there are services promoted as “we’re there for you 24/7” even though...there’s always reasons where things fail and we’re used to that. For some reason...I’m surprised how devastated I was that ...I had this 45 year old guy...diabetic type 1 who was having this kind of rumbling myocardial infarction and going into heart failure...and I was like I’m gonna call RUDi and they’re gonna help me get him out of here and then nobody answered and then it was like ahhhh now I have to call [PTN]...I think it’s not a failure of RTVS but it’s just kind of one of the drawbacks of any service…So that’s one danger of I think any service is how sustainable is it. (Participant #15)

This highlights the importance participants place on sustaining RTVS; for these rural providers, RTVS has become embedded in their practice.

##### Theme 3: Continuity of Care

Another challenge raised by a few participants was the need for integration of RTVS support into the health system for continuity of care for patients. One community health nurse explained:

The challenges would be that these doctors aren’t seeing these patients regularly. There’s not great follow-up. It’s not really appropriate to have them prescribing any long-term medications. And there’s a bit of a disconnect between handing off these patients to a GP if these patients are unattached. (Participant #1)

##### Theme 4: Mental Health Service Gaps

The above challenge of continuity was highlighted in relation to gaps in service for mental health in their community. Findings support the importance of integration of RTVS into the local context as part of hybrid care, which could contribute to addressing this gap.

If there was a patient that came in with a situational crisis or mental health crisis or...domestic abuse...and requires local involvement as well, I would say the RTVS struggles a little bit but of course like it’s a virtual consultation support and they don’t live here, they don’t have the full local context...that requires a more of a team to approach I would say is a bit more challenging for the RTVS to handle as a primary need. I don’t have a solution for that. (Participant #11)

Findings underscore the importance of integration of supports such as RTVS into primary care in RRFNI communities, to enhance continuity and avoid fragmentation of care.

## Discussion

HCPs working in RRFNI communities face unique challenges. These challenges include geographic isolation, burnout, issues with recruitment and retention of health workforce on site, and technology infrastructure gaps.^10^ The RTVS program represents a novel approach to addressing some of these challenges by integrating virtual physician peer support services into the health system in RRFNI communities.^19^ In fiscal year 2022/2023, 118 distinct communities across BC accessed the RTVS peer pathways.^19^ The three peer RTVS pathways reported that more than 35% of the communities served are First Nations and other Indigenous communities (35.4% for CHARLiE, 41.9% for MaBAL, and 41.6% for RUDi).^19^ The high utilization of the program highlights its potential benefits for these communities, which historically face inequities in access to healthcare.

HCPs in our analysis emphasized timely clinical support, enhanced collegiality, and increased confidence in practice resulting from RTVS peer pathways. These benefits reflect LeBlanc et al.^24^ who found that telehealth initiatives have significantly improved access to healthcare for both patients and providers, though in different ways. While patients gained increased access to physicians and specialists through telehealth, healthcare providers, particularly nurses, benefited from access to diverse clinical support services. A key benefit highlighted in our study was timely access to support for HCPs, especially during overnight or high-demand periods, reinforcing our findings related to professional connection and reduced anxiety in isolated practice settings.

Our findings suggest that virtual peer support contributes to HCP retention in rural and remote settings. This is consistent with the work of Wieland and colleagues,^4^ who noted that the presence of experienced colleagues providing mentorship and peer support plays a crucial role in mitigating stress among rural GPs. The absence of such collegial support has been linked to increased work-related distress and a higher likelihood of practitioners considering leaving remote practice.^5^ These findings underscore the importance of peer support systems not only for provider well-being but also for long-term workforce sustainability.

The findings from this study contribute to a growing body of evidence supporting RTVS as an effective strategy for strengthening healthcare in RRFNI communities. This study offers an understanding of how RTVS is experienced by frontline HCPs, complementing previous evaluations focused on service utilization and patient outcomes.^19^ Insights gathered here informed the ongoing expansion of the RTVS program, including provider retention and recruitment in RRFNI communities and the addition of new pathways.

Together, these findings suggest that RTVS improves access to care for RRFNI communities. Meanwhile, having access to VPs reduces the clinical burden on rural HCPs and improves their confidence by offering collegial support. Findings from this study indicate that healthcare providers in RRFNI communities consider the RTVS program and its VPs to be delivering high-quality support. Healthcare providers described VPs as approachable, responsive, respectful, and understanding of the rural healthcare context. This echoes the broader evaluation literature, where VPs were described highly for interpersonal and clinical competencies, interprofessional collaboration, and understanding of Indigenous and rural contexts.^19^

Despite the advantages, participants also reported some challenges, including technological disruptions, administrative burdens, and gaps in mental health services. The technological concerns were infrequent but mirror those documented by LeBlanc et al.^24^ who noted that systemic barriers, particularly unreliable technology and lack of high-speed Internet in rural areas, remain significant obstacles to effective virtual care delivery. These limitations provided in-depth and contextualized feedback that informed the development and expansion of RTVS to increase coverage and decrease uncertainty of not getting a hold of RTVS VPs, as reflected in our results.

## Strengths and Limitations

Strengths include understanding the experiences of rural and remote HCPs providing care in RRFNI communities through in-depth interviews. These HCPs’ experiences contributed to the overarching evaluation of RTVS that shed light on the outcomes of how access to RTVS peer support is increasing access to healthcare in RRFNI communities. These findings should be interpreted in light of certain limitations. One limitation is related to the participants interviewed excluded HCPs who did not use RTVS. This limits the potential for understanding the experiences between users and non-users of the RTVS peer pathways in RRFNI communities. However, RUDi, CHARLiE, and MaBAL supported 57% of the 147 high-priority, edge communities,^19^ which represents a large coverage of the RSA-A communities and speaks to the significance of experiences gathered from this participant group. Another limitation is that only one midwife participated in the evaluation, which limits our ability to understand the unique experience of this provider group. Future studies should put emphasis on recruiting more midwives so as to gain an in-depth understanding of MaBAL usage by midwives in RRFNI communities. Furthermore, the interview guide may have implicitly directed participants towards specific themes or areas of inquiry. While this is an inherent challenge in qualitative research, our study used an open-ended semi-structured interview guide to help address this limitation. Reflexivity is another important consideration, as the team members’ prior experience with the healthcare system and virtual care may have influenced question framing in the interview guides and interactions with participants during these interviews. Lastly, our findings represent the experiences and BC context of the HCPs interviewed which should be considered when applying our findings to broader communities and would benefit from future expansion of feedback.

## Conclusion

Our findings highlight the importance of RTVS in supporting HCPs and providing more equitable access to care in RRFNI communities. Several implications of this study are highly relevant to policy and leadership: first, providing timely virtual support as an essential service for HCPs working in RRFNI communities. The MoH identified a key goal in their 2025-2026 - 2027-2028 service plan for BC to have “a high-quality sustainable health system supported by a skilled and diverse workforce, and effective and efficient systems and structures.”^25^ This goal includes implementing strategies to recruit and retain providers and technology use for facilitating equitable service delivery.^25^ Providers working in isolation or with limited clinical support in RRFNI communities would be more likely to be recruited and retained with appropriate clinical support provided through RTVS. The second implication is the importance of enhancing and maintaining technological infrastructure in RRFNI communities, so HCPs and their patients have equitable access to healthcare through virtual care. Third, sustained funding for RTVS is essential as this program is integrated in the fabric of RRFNI communities where HCPs rely on being able to “phone a friend” to provide culturally safe and high-quality care for patients. Finally, it is vital to explore the impact of RTVS on rural and remote HCPs’ well-being and access to mental health support for their patients. RTVS peer support is essential for rural HCPs and has become a standard of practice, as a unique and valuable way for equity of access to high-quality care, and support for health professional experiences, recruitment, and retention.

## Data Availability

The data gathered and analyzed during the study are not publicly available to protect the participants' confidentiality.*
